# Empowered to Break the Silence: Applying Self-Determination Theory to Employee Silence

**DOI:** 10.3389/fpsyg.2019.00485

**Published:** 2019-03-05

**Authors:** Dong Ju, Li Ma, Run Ren, Yichi Zhang

**Affiliations:** ^1^Business School, Beijing Normal University, Beijing, China; ^2^Guanghua School of Management, Peking University, Beijing, China

**Keywords:** employee silence, empowering leadership, self-determination theory, job autonomy, intrinsic motivation

## Abstract

The paper studies how leaders can break employee silence. Drawing upon self-determination theory, we argue that empowering leadership can activate employees’ intrinsic motivation such that employees are more willing to break the silence at work; furthermore, the effect is stronger when employees have high (vis-à-vis low) levels of job autonomy. We collected time-lagged and multi-source data in a large company to test our hypotheses. The results show that intrinsic motivation mediates the relationship between empowering leadership and employee silence. That is, empowering leadership can reduce employee silence through enhancing their intrinsic motivation. Furthermore, this mediation effect will be stronger when employees have high levels of job autonomy. This paper contributes to the literature on leadership, employee silence, and job design characteristics.

## Introduction

Employees operating on the front line know best about how their organizations can improve, but they often keep silent about their opinions (Tangirala and Ramanujam, 2008a). A study indicates that over 85% of the managers and professionals interviewed admitted that they had kept silent about work concerns (Milliken et al., 2003). Employee silence is defined as “the withholding of ideas, suggestions, or concerns about people, products, or processes that might have been communicated verbally to someone inside the organization with the perceived authority to act” (Kish-Gephart et al., 2009, pp 166–167). When employees keep silent about potential improvements to their organizations, they may feel unsatisfied and uncommitted; their leaders will not be able to obtain useful information, ideas, and opinions; and their organizations may be in danger of stagnation (Argyris and Schon, 1978; Glauser, 1984; Dutton and Ashford, 1993; Argote and Ingram, 2000; Morrison, 2011). In contrast, if employees frankly present their concerns and suggestions about possible changes to work activities, their organizations can enjoy improved work processes and innovation (Argyris and Schon, 1978; Glauser, 1984) and higher levels of organizational performance (Argote and Ingram, 2000).

Among the numerous factors that affect employee silence is the salient role of leaders who help define the working context around employees. Leaders play a key role in defining employees’ working behaviors (Yukl, 1994). In this paper, we study how leaders can effectively reduce employee silence by empowering them. Empowering leadership can enable employees to enjoy the feeling of being a valued team member in their work and to feel that they are insiders who are competent and valued by their leaders (Ahearne et al., 2005). We believe that all of these mechanisms help break employee silence.

Keeping silent is an easy choice for employees because the lack of action often represents the status quo and satisfies their extrinsic needs (Premeaux and Bedeian, 2003). Breaking the silence, however, requires greater energy and motivation such that employees are willing to break the silence only when they have intrinsic motivations to do so. We focus on employees’ intrinsic motivation about their jobs to study the effect that empowering leadership can break employee silence. Following the guidance of self-determination theory (SDT), we study empowering leadership rather than other leadership styles such as servant leadership (which can also empowers subordinates to speak up; Linuesa-Langreo et al., 2018) because empowering leadership can increase subordinates’ feelings of autonomy, relatedness, and competence that can enhance their intrinsic motivation.

In addition, this effect tends to be stronger when the work is designed with a high rather than low level of job autonomy. We build a model linking empowering leadership and employee silence with employees’ intrinsic motivation at work as the mediator of this process, and with job autonomy serving as the moderator of the empowering leadership–intrinsic motivation relationship. The model reveals the underlying mechanism and the boundary condition of why and how empowering leadership can help break employee silence. To test the proposed hypotheses, we collected time-lagged multi-source data in a large multi-national electronics group operating in China.

The research contributes to management theory and practice in three aspects. First, we incorporate SDT to understand how to break employee silence. A lack of motivation is the most important reason for employee silence (Morrison, 2011). SDT focuses on people’s motivation to develop their potential and personal growth, not on minimizing the costs to obtain rewards and pleasure (Sheldon et al., 2003; Gagné and Deci, 2005). Expressing ones’ recommendations and concerns to leaders is not required in job descriptions, and can even induce negative outcomes (Van Dyne and LePine, 1998). Thus, intrinsic motivation is necessary for breaking silence.

Second, most of the literature linking leadership and silence is based on the logic that leadership can create or eliminate a safe environment for employees to express their opinions (Detert and Burris, 2007; Walumbwa and Schaubroeck, 2009; Walumbwa et al., 2012), or on the reciprocal norm that leaders can encourage employees to speak out for the benefit of the teams and the organization (e.g., Burris et al., 2013). However, this line of research does not pay enough attention to the importance of leaders’ role in enhancing employees’ intrinsic motivation to speak up for their work performance, which is the gap that this paper fills. Based on SDT, we examined the effects of empowering leadership on employee silence via intrinsic motivation. Empowering leadership allows employees to work with delegation and confidence. Even when the leader and the subordinate have different ideas, they can work together on solutions that are beneficial to the work unit (Ahearne et al., 2005). Empowering leadership shows sympathy and concern for the employees and connects them to the whole team. As SDT suggests, all of these positive effects will enhance employee intrinsic motivation.

**FIGURE 1:**
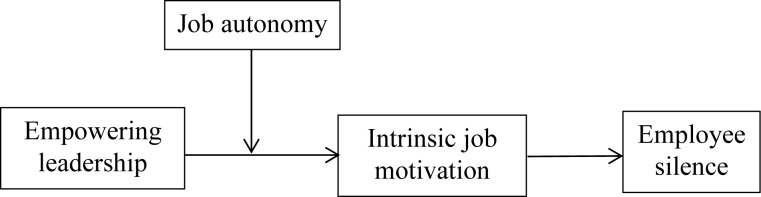
Theoretical framework.

Third, we emphasize the importance of job autonomy as a supporting context for enhancing empowering leadership’s effects on increasing intrinsic motivation and reducing employee silence. It would be shortsighted to assume leadership’s effect on employee behavior without considering the influence of work design (Hackman and Oldham, 1976). In line with SDT, job autonomy represents autonomous support in the work context. Thus, the beneficial effects of empowering leadership on employee intrinsic motivation and then on employee silence also depend on the supporting context—in our study, job autonomy. Thus, we adopt the moderated mediation hypothesis to comprehensively analyze how and when empowering leadership can break employee silence (Figure 1).

### Theory and Hypotheses

#### Self-Determination Theory and Individual Behaviors

Individuals engaging in conscious actions can demonstrate intrinsic and/or extrinsic motivation (Gagné and Deci, 2005). Extrinsic motivation is based on the anticipation that the action can bring something the actor pursues, such as a reward or status (Deci and Ryan, 1985). In comparison, intrinsic motivation is “based in the innate, organismic needs for competence and self-determination” (Deci and Ryan, 1985, p. 32). Intrinsic motivation centers on SDT and differentiates SDT from previous motivational theories. SDT assumes that all individuals have three universal and evolved needs—autonomy, competence, and relatedness—and these three needs foster intrinsic motivation and internalization (e.g., Baumeister and Leary, 1995). When the three needs are satisfied, individuals are motivated by their own internal nature to perform. Autonomy means the need “to be self-regulating, to be the maker or at least the owner of one’s choices” (Sheldon et al., 2003, p. 366). Competence means the need “to be effective in what one does, mastering new skills in the process” (Sheldon et al., 2003, p. 366). Finally, relatedness means the need “to feel connected and in sympathy with at least some others” (Sheldon et al., 2003, p. 366).

While individuals differ in terms of the strengths of these three types of needs, SDT focuses on the fact that all individuals possess these needs. Consequently, the satisfaction of these needs will enhance individuals’ intrinsic motivation to engage in the activity. When the work environment supports the satisfaction of psychological needs, it will promote employees’ intrinsic motivation for their tasks. For example, leaders who help followers develop their interests and appeal to their collective identities can encourage improved employee intrinsic motivation and job performance (Shamir et al., 1993).

#### Empowering Leadership and Employee Silence

Although speaking up can contribute to the well-being of the organization, breaking silence in organizations is both voluntary and risky, which may cause employees to keep silent (Ashford et al., 1998; Van Dyne and LePine, 1998; Morrison and Milliken, 2000; LePine and Van Dyne, 2001; Detert and Burris, 2007). First, breaking silence is voluntary because it is not (and often cannot be) required by a job description (Van Dyne and LePine, 1998; LePine and Van Dyne, 2001); the action is beyond the role requirement and thus, by definition, a type of extra-role behavior (Van Dyne and LePine, 1998; LePine and Van Dyne, 2001). Employees who keep silent are thus not scrutinized or punished for choosing not to proactively offer their valuable recommendations and ideas to their organizations. Second, breaking silence is risky because employees who voice their views sometimes receive backlash, both cognitively (e.g., they are seen as less attractive or hostile) and behaviorally (e.g., they receive less favorable performance appraisals) (Detert and Edmondson, 2011). The risk associated with voice deters many employees from expressing their ideas at all (Detert and Burris, 2007).

These two factors of breaking silence are reflected in employees’ cognitive understandings of the reasons that they remain silent when they have potentially valuable ideas for the operation of the organization. Exploratory studies found that employees keep silent in organizations for multiple reasons. For example, Detert and Edmondson (2011) used the “lay theory” (implicit belief or implicit theory) approach and found that employees keep silent because of five beliefs: their leaders may feel challenged, they need solid data or solutions (to speak up), they do not want to make the boss feel embarrassed in front of higher level officers, they do not want to embarrass the boss in public, and they do not want to incur negative career consequences from speaking up (Detert and Edmondson, 2011). These reasons are effective, possibly because employees do not feel a strong innate drive to break their silence (Detert and Trevino, 2010).

According to SDT, empowering leadership can encourage employees to break their silence for three reasons. First, empowering leadership can “unleash employees’ potential, enhance their motivation, allow them to be more adaptive and receptive to their environment, and minimize bureaucratic hurdles that slow responsiveness” (Ahearne et al., 2005, p. 945). Empowering leadership increases employees’ feelings of responsibility in their jobs through delegation and discretion. Thus, the felt obligation to improve their organizations drives the employees to break the convenient choice of keeping silent.

Second, empowering leadership encourages employees to believe that they are capable and that they have the right to offer their concerns and suggestions. Conger and Kanungo (1988) suggested that empowerment enables employees to believe that their contributions are closely connected to the organization’s goals. Integrating Bandura (1986) self-efficacy notion, empowering leadership can be defined as behaviors that enable employees to believe in their self-efficacy or to perform a specific task. For example, empowering leaders make employees believe that they can make upward communications about important work issues and encourage them to deliver their ideas regarding working more effectively rather than remaining silent.

Third, the behaviors of empowering leadership are necessary to support employees’ need for relatedness. Empowering leadership was first conceptualized as an aspect of the relational view (Burke, 1986; Burpitt and Bigoness, 1997) that originated from the Ohio State leadership studies on showing concern for subordinates (Fleishman, 1953). Empowering leadership encourages team members to communicate ideas and information and to make suggestions to increase formal and informal interactions between team members (Srivastava et al., 2006). Empowering leadership encourages cooperation to accomplish collective goals and promotes understanding among team members through persistent appeals for collaboration, which can help employees establish harmonious relationships with other coworkers and make them feel connected (Carmeli et al., 2011; Lee et al., 2018). The feelings of being related to their supervisors and organizations make them willing to take the risk related to expressing their opinions. In other words, breaking silence represents their willingness to contribute to the organizations and groups they identify with. In sum, empowering leadership encourages employees to take initiative on the job by offering them discretion, helps employees to feel competent by expressing confidence in their abilities, and makes employees feel related to the work group/organization by showing sympathy and concern for their feelings and thoughts. All of these positive effects form as the driving force for easing employees from withholding important issues for the work by being empowered by their leaders.

Hypothesis 1: Empowering leadership is negatively related to employee silence.

#### The Mediating Role of Intrinsic Motivation

Among the factors that keep employees silent, a lack of motivation is the most important (Morrison, 2011). When employees are not intrinsically motivated to engage in risky extra-role behavior (i.e., voice), they simply keep silent (e.g., Detert and Edmondson, 2011). Intrinsic motivation at work offers the strong basic drive for employees to behave proactively and productively (Deci and Ryan, 1985). Intrinsic motivation encourages employees to invest more of their energy, attention, and time in their work, such that their performance tends to improve (Zapata-Phelan et al., 2009). In addition, intrinsic motivation is a strong predictor of employee creativity (Zhang and Bartol, 2010), which helps to develop ideas for improving the function of the organization. Intrinsically motivated employees work toward their personal achievements, and the success of their tasks can provide them with intrinsic satisfaction (Warr et al., 1979). They can obtain subjective rewards associated with their self-esteem by performing well for the organization (Lawler, 1969). To some extent, their needs for intrinsic satisfaction can outweigh the perceived risk of breaking their silence. Ruh et al. (1975) reported that employees who intrinsically work hard will view their job as the central part of their lives and will be more likely to participate in decision making. With intrinsic motivation, employees tend to have higher levels of creativity, concentration, initiative, and flexibility (Thomas and Velthouse, 1990), which are the key qualities for employees’ willingness to offer beneficial suggestions. In addition, intrinsically motivated employees have strong attachments to their jobs and focus on high levels of performance (Zapata-Phelan et al., 2009). Their drive to excel may stop them from being silent on work matters that might negatively impact their jobs.

According to our above analyses guided by SDT, empowering leadership increases employees’ intrinsic motivation. Intrinsic motivation at work can be activated when employees feel that they are competent, autonomous, and related to their leaders. Satisfying these needs can enhance employees’ intrinsic motivation, and motivation was emphasized as the most important factor for breaking the silence (Morrison, 2011). Empowering leadership can unleash employees’ potential and encourage them to self-initiate and pursue higher job satisfaction (Deci et al., 1989). Leaders can increase employees’ intrinsic motivation (Deci et al., 1989). First, employees will feel that they have more autonomy and discretion when their leaders empower them to make decisions at work. A great amount of evidence indicates that autonomy support helps maintain and enhance intrinsic motivation (Ryan and Stiller, 1991). Second, empowering leadership signals to employees that their competence is satisfactory to their leaders. Empowerment grants a certain amount of discretion to employees to make decisions relevant to their jobs. The responsibilities associated with this discretion require the employees to be empowered to use their competence wisely. Empirical research indicates that empowerment increases employees’ feelings of competence (Peccei and Rosenthal, 2001).

Third, empowering leadership fosters employees’ feelings of relatedness to their leaders. Empowerment grants the discretion to employees, but the leaders also need to take the risk of the empowerment. Obviously, leaders’ empowerment actions are made under the presumption that the employees will act in the direction intended by the leaders. This empowerment signals to the employees that their leaders trust them and believe that their relationships are close (van Dijke et al., 2012). Taken together, these aspects of empowering leadership foster employees’ intrinsic motivation. Empirical studies have also found that when leaders empower employees, these employees tend to be more satisfied in terms of their needs for competence, relatedness, and autonomy (Kasser et al., 1992; Ilardi et al., 1993; Gagné et al., 2000; Deci et al., 2001; Baard et al., 2004).

Following this line of logic, we argue that intrinsic motivation at work will drive employees to more deeply engage in actions to improve their work settings and performance and encourage them not to withhold their valuable suggestions from their supervisors. As Morrison (2011) review paper suggested, motivation can strongly reduce employees’ silence behavior. In summary, empowering leadership can fulfill employees’ needs for autonomy, competence, and relatedness, all of which help establish intrinsic motivation in their jobs. Working with intrinsic motivation enhances employees’ feeling of responsibility for the well-being of the whole work unit and the organization, which decreases employee silence.

Hypothesis 2: Intrinsic motivation mediates the relationship between empowering leadership and employee silence.

#### Job Autonomy as Supporting Context

Job autonomy increases employees’ feeling that they are capable, autonomous, and related to their supervisors. Job autonomy is “the degree to which the job provides substantial freedom, independence, and discretion to the individual in scheduling the work and in determining the procedures to be used in carrying it out” (Hackman and Oldham, 1976, p. 258).

Job autonomy strengthens the effect that empowering leadership has on intrinsic motivation for three reasons. First, job autonomy enables empowering leadership to strengthen employees’ sense of control and autonomy. Job autonomy offers employees the freedom to determine their daily time plans, to take initiative, and to make judgments in carrying out the work (Deci et al., 1989; Spreitzer, 1995). All of these factors enable employees to use the empowerment received from their leaders. When employees are given the expectations of empowering leadership to take initiative, if the working context also allows them to control the work process, they will receive more autonomous support from the job, which will lead them to pursue more intrinsic satisfaction from the job. In contrast, when job autonomy is low, the empowerment from their leaders has little ability to enable employees to schedule their own plans or to put their own ideas into practice. As a result, the employees will not feel that they are capable of conducting the job. In such cases, the relationship between empowering leadership and intrinsic motivation tends to be weaker.

Second, job autonomy provides employees with an ideal context in which to take full advantage of their abilities at the job (Morgeson et al., 2005), which strengthens the effect of empowering leadership. Employees with high job autonomy have various means to promote their jobs (Wang and Netemeyer, 2002; Morgeson et al., 2005; Wang and Cheng, 2010). This magnified sense of competence obtained from both the leaders and the job context will motivate employees to derive satisfaction from task accomplishment and therefore work harder to excel rather than to withhold critical information and suggestions.

Third, job autonomy enhances the effect of empowering leadership on employees’ sense of belonging and satisfies their need for relatedness. Employees are intrinsically motivated to work when leaders show concern for them and help them relate to other people (van Dijke et al., 2012). This effect is magnified when job autonomy offers them sufficient discretion to best work toward the work unit goal and then contribute to the welfare of others with whom they are connected.

Hypothesis 3: Job autonomy moderates the strength of the mediated relationship between empowering leadership and silence via intrinsic motivation such that the mediated relationship will be stronger under high job autonomy than under low job autonomy.

## Materials and Methods

### Procedures and Participants

Participants were several 1000 full-time employees in 19 companies of a large multi-national electronics group operating in China. The employees worked for multiple departments at various levels and were based in different cities. As part of the consulting service offered to the company, the company’s HR division randomly assigned each participant an ID number, which participants included on their surveys. This design ensured accurate matching between the two surveys and the archival data and the anonymity of the responses.

The two surveys were completed online 2 weeks apart. At time 1, participants reported their direct supervisors’ empowering leadership and their job autonomy. Two weeks later (time 2), participants reported their intrinsic motivation and silence. In total, 8,079 participants completed the time 1 survey, 5,169 completed the time 2 survey, and 3,717 responses were matched between time 1 and time 2. We followed the procedures recommended by Goodman and Blum (1996) in performing a logistic regression to assess the potential effect of attrition. The results indicated no difference between time 1 and time 2 (the results of the logistic regression analyses are available upon request from the first author).

The final sample consisted of 3,717 employees from 545 groups. The majority were under 30 years old (51.44%), and 27.44% were between 30 and 35 years old. The average organizational tenure was 4.97 years, and tenure ranged from 1 to 21 years (*SD* = 3.87). The majority (68.21%) of the employees had a bachelor’s degree, and 70.17% were male. The average number of employees in each group is 6.82.

### Measures

We translated the scales that were originally in English into Chinese and checked the equivalence using the back translation approach (Brislin, 1980). Unless otherwise indicated, all of the measurements were presented in a six-point Likert format (1 = strongly disagree; 6 = strongly agree).

#### Silence

Silence was measured using the five-item scale used by Tangirala and Ramanujam (2008a). An example item is “Although I had ideas for improving work effectiveness in my organization, I did not speak up” (α = 0.90).

#### Intrinsic Motivation

We assessed this construct with six-item scale developed by Warr et al. (1979), which was also used by Bond et al. (2008). An example item is “I take pride in doing my job as well as I can” (α = 0.89).

#### Empowering Leadership

We measured empowering leadership using a scale from Ahearne et al. (2005). This scale has four subscales that emphasize (a) enhancing the meaningfulness of work, (b) fostering participation in decision making, (c) expressing confidence in high performance, and (d) providing autonomy from bureaucratic constraints. Example items of each subscale are as follows: “My manager helps me understand how my objectives and goals relate to that of the Company,” “My manager makes many decisions together with me,” “My manager believes that I can handle demanding tasks,” and “My manager allows me to do my job my way.” In our study, we adopted the back-translation (Brislin, 1980) procedure to make sure that the Chinese version of the scale creates identical meaning to our participants. In this procedure, we found that two of the items out of the first dimension (i.e., Enhancing the meaningfulness of work) were exactly the same when they were translated into Chinese. Thus, in line with previous research (e.g., Yam et al., 2017), we deleted one of them. Combining all of the items, the overall scale of empowering leadership had a satisfactory reliability coefficient (α = 0.94).

#### Job Autonomy

Autonomy was measured with the three-item scale of Idaszak and Drasgow (1987) for assessing work characteristics. An example item is “The job gives me considerable opportunity for independence and freedom in how I do the work” (α = 0.87).

#### Control Variables

We controlled participants’ gender, age, organization tenure, and education, as demographic variables have been shown to influence employee silence (cf. Detert and Edmondson, 2011).

## Results

Descriptive statistics, bivariate correlations, and Cronbach’s alphas for all of the variables are presented in Table 1. Empowering leadership was positively related to intrinsic motivation (*r* = 0.43, *p* < 0.01) and negatively related to employee silence (*r* = −0.25, *p* < 0.01). Intrinsic motivation was negatively correlated to employee silence (*r* = −0.22, *p* < 0.01). These bivariate results provided preliminary support for the hypotheses.

**Table 1:** Descriptive statistics and correlations.

	*M*	*SD*	1	2	3	4	5	6	7
(1) Sex (1-male, 2-female)	1.30	0.46	–						
(2) Age	(0.00)	0.94	−0.094^∗∗^	–					
(3) Education	(0.00)	0.72	−0.017	0.040^∗^	–				
(4) Tenure	(0.05)	3.87	0.029	0.632^∗∗^	0.048^∗∗^	–			
(5) Silence	(0.02)	0.89	−0.046^∗∗^	−0.068^∗∗^	0.031	−0.080^∗∗^	–		
(6) Intrinsic motivation	0.00	0.66	0.055^∗∗^	0.067^∗∗^	−0.088^∗∗^	−0.009	−0.217^∗∗^	–	
(7) Autonomy	0.01	1.01	0.025	0.045^∗∗^	−0.067^∗∗^	0.058^∗∗^	−0.199^∗∗^	0.324^∗∗^	–
(8) Empowering leadership	(0.00)	0.95	−0.020	0.031	−0.060^∗∗^	−0.006	−0.247^∗∗^	0.426^∗∗^	0.532^∗∗^

*^∗∗^Correlation is significant at the 0.01 level (two-tailed). ^∗^Correlation is significant at the 0.05 level (two-tailed).*

Confirmatory factor analysis with empowering leadership, intrinsic motivation, job autonomy, and employee silence showed that the model fits the data well (χ^2^ = 5523.70, *df* = 224, *p* < 0.001; RMR = 0.05, RMSEA = 0.08, CFI = 0.95, IFI = 0.95), providing evidence for the validity of the measures.

### Main Effects

Due to the nested nature of our data collection, we conducted hierarchical linear modeling (HLM) (Bryk and Raudenbush, 1992) to test all of the hypotheses. We first ran a one-way analysis of variance with random effects. The null model (Model 1 in Table 2) revealed that there was significant variance between groups with respect to intrinsic motivation: τ_00_ = −0.02, and the Chi-square test comparing model 1 and the corresponding linear regression model (which does not consider the hierarchical structure) showed that model 1 was significantly better than the linear regression model [χ^2^(545) = 28.51, *p* < 0.001]. Hypothesis 1 stated that empowering leadership was negatively related to silence, and the results of Model 8 supported this hypothesis (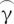 = −0.23, *p* < 0.001).

**Table 2:** HLM results predicting intrinsic motivation and silence.

	**Model 1**	**Model 2**	**Model 3**	**Model 4**	**Model 6**	**Model 7**	**Model 8**	**Model 9**	**Model 10**
		
	Dependent variable = Intrinsic motivation				Dependent variable = Silence				
Intercept	−0.02	−0.13^∗∗∗^	−0.14^∗∗∗^	−0.16^∗∗∗^	−0.02	0.10^∗^	0.11^∗^	0.08†	0.07†
Age		0.09^∗∗∗^	0.07^∗∗∗^	0.08^∗∗∗^		−0.04†	−0.02	−0.01	−0.01
Sex		0.09^∗∗∗^	0.11^∗∗∗^	0.10^∗∗∗^		−0.09^∗∗^	−0.10^∗∗^	−0.08^∗∗^	−0.07^∗^
Education		−0.07^∗∗∗^	−0.06^∗∗∗^	−0.05^∗∗∗^		0.04^∗^	0.02	0.01	0.01
Tenure		−0.01^∗∗∗^	−0.01^∗∗∗^	−0.01^∗∗∗^		−0.01^∗∗^	−0.02^∗∗^	−0.02^∗∗∗^	−0.01^∗∗^
Empowering leadership			0.29^∗∗∗^	0.25^∗∗∗^			−0.23^∗∗∗^	−0.18^∗∗∗^	
Autonomy				0.10^∗∗∗^					−0.12^∗∗∗^
Autonomy × Empowering leadership				0.04^∗∗∗^					
Intrinsic motivation								−0.18^∗∗∗^	−0.24^∗∗∗^
Intrinsic motivation × Autonomy									−0.04^∗^
N (Level 1)	3717	3717	3717	3717	3717	3717	3717	3717	3717
N (Level 2)	545	545	545	545	545	545	545	545	545
Model deviance^a^	7459.51	7399.08	6672.72	6583.55	9696.61	9658.12	9424.54	9367.16	9411.98

*^a^Deviance = −2 × log-likelihood of the full maximum-likelihood estimate, which is a measure of model fit – the smaller the deviance is, the better the model fits. ^†^p < 0.10, ^∗^p < 0.05, ^∗∗^p < 0.01, ^∗∗∗^p < 0.001.*

### Indirect Effects

Hypothesis 2 proposes that empowering leadership will have a negative indirect relationship with silence through intrinsic motivation. We adopted RMediation technique to test the indirect effect (Tofighi and MacKinnon, 2011), which has become an emerging method for testing mediation (e.g., Rodell, 2013). This technique builds confidence intervals for mediated effects based on methods such as the distribution of the product. This technique is superior for testing mediation because it has more accurate type I error rates than alternative methods, and better statistical performance than the traditional Sobel test (MacKinnon et al., 2004, 2007). The indirect effect is calculated by multiplying the path coefficient from empowering leadership to intrinsic motivation (*b* = 0.29) by the path coefficient from intrinsic motivation to silence (*b* = −0.18), and the result is significant when submitted to the RMediation test (indirect effect = −0.052, *SE* = 0.006, *p* < 0.01).

### Moderated Mediation

Hypothesis 3 was a moderated mediation proposing that the relationship between empowering leadership and silence via intrinsic motivation was stronger when job autonomy was high. Testing moderated mediation with the path analytic method has been shown to have the greatest statistical performance (MacKinnon et al., 2002). In addition, bootstrapping methodologies should be used to test the statistical significance of indirect effects in moderated mediation models (Edwards and Lambert, 2007). This paper followed the path analytic procedures proposed by Edwards and Lambert (2007), which has been widely applied by previous research (e.g., Qin et al., 2018). The procedures involved estimating the following two equations, representing the test for a first- and second-stage moderated mediation model.

Intrinsic motivation = *a*_0_ + *a*_1_empowering leadership + *a*_2_autonomy + *a*_3_empowering leadership × autonomy + *e* (1)

Silence = *b*_0_ + *b*_1_empowering leadership + *b*_2_intrinsic motivation + *b*_3_autonomy + *b*_4_intrinsic motivation × autonomy + *e* (2)

**FIGURE 2:**
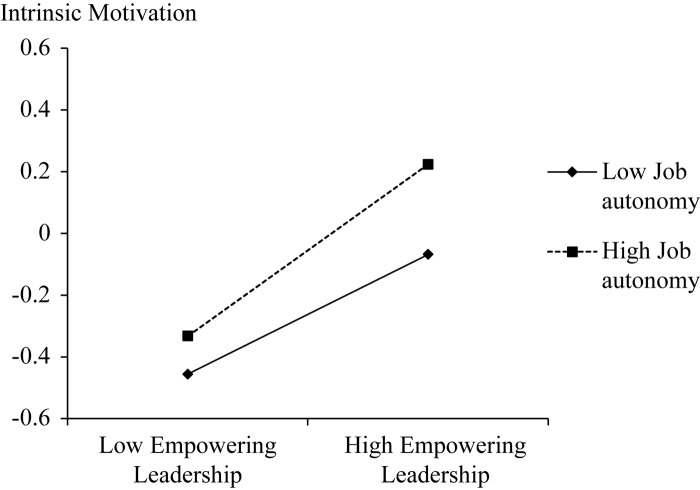
Interactional effect of empowering leadership and job autonomy on intrinsic motivation.

We substituted Equation (1) for intrinsic motivation into Equation (2) and calculated the indirect effects at low and high levels of autonomy. We bootstrapped 1,000 samples to obtain bias-corrected confidence intervals to test the significance of the indirect effects. The results of hypothesis 2 support the mediation effect of intrinsic motivation on empowering leadership and employee silence. We adopted regression to test the moderating effect of job autonomy on the relationship between empowering leadership and intrinsic motivation. We entered empowering leadership, job autonomy, and empowering leadership × job autonomy in Model 4 in Table 2. As expected, the product of empowering leadership and job autonomy was significant (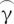 = 0.04, *p* < 0.001). We also plotted this moderating effect to fully understand the nature of moderation (Figure 2). In accordance with Cohen et al. (2013), high and low levels of moderation were used at +1 and −1 standard deviation from the mean of the moderator variable. A simple slope test showed that the relationship of empowering leadership and intrinsic motivation was stronger when job autonomy was high rather than low (high job autonomy: *B* = 0.15, *p* < 0.001; low job autonomy: *B* = 0.06, *p* < 001).

Further, we presented the results of path analysis in Table 3. For low job autonomy (i.e., one standard deviation below the mean), the first- and second-stage simple effects are 0.21 (*p* < 0.01) and −0.15 (*p* < 0.01), respectively. Thus, the indirect effect for low job autonomy is −0.03 (*p* < 0.01). For high job autonomy (i.e., one standard deviation above the mean), the first- and second-stage simple effects are 0.30 (*p* < 0.01) and −0.22 (*p* < 0.01), respectively. Hence, the indirect effect for high job autonomy is −0.07 (*p* < 0.01). Differences in the effects for low and high job autonomy are significant at the first stage (0.09, *p* < 0.01), but not at the second stage (−0.06, *n.s.*). The differences for indirect effects are also significant (−0.03, *p* < 0.01). In conclusion, these results support hypothesis 3. We also reported a full model of hypothesis testing (Model 10 in Table 2), and the results remained virtually the same, indicating the robustness of our results reported above.

**Table 3:** Results of the moderated path analysis.

Moderator variable	Stage effect				
	First	Second	Direct	Indirect	Total
	Empowering leadership → Intrinsic motivation → Silence				

Low autonomy	0.21^∗∗^	−0.15^∗∗^	−0.14^∗∗^	−0.03^∗∗^	−0.18^∗∗^
High autonomy	0.30^∗∗^	−0.22^∗∗^	−0.14^∗∗^	−0.07^∗∗^	−0.21^∗∗^
Differences between low and high autonomy	0.09^∗∗^	−0.06	−0.00	−0.03^∗∗^	−0.03

*^∗∗^p < 0.01.*

## Discussion

Employees sometimes keep silent about critical work issues and are reluctant to offer suggestions. However, scholars and practitioners are devoted to minimizing employee silence and to finding effective ways to motivate employees to offer beneficial suggestions. The central objective of this paper was to examine how and when leadership can reduce employee silence. In a survey over a 2-week period of a multi-national company, the main effect results demonstrated that empowering leadership is associated with increased intrinsic motivation, which is in turn related to decreased employee silence. The findings also indicated that job autonomy strengthens the positive relationship between empowering leadership and intrinsic motivation and the indirect relationship between empowering leadership and employee silence.

### Theoretical Contributions

This paper advances the silence literature in several ways. First, the literature on silence/voice has drawn upon relatively weak theory to explain these behaviors, especially about the effects of leadership behaviors on silence (Morrison, 2011). Previously, when studying leadership and silence/voice, many scholars have argued from the perspective of psychological safety or identification. These lines of research based on leadership can reduce employees’ perceived risk and enhance the benefits that they can receive from speaking up (Detert and Burris, 2007), or employees are identified with the leader (Liu et al., 2010), causing them to share potentially important information with others. However, because employees’ silence/voice is a self-determined behavior, applying SDT to the silence/voice literature offers a new perspective to examine employee silence/voice behavior. This paper firstly integrates SDT into the voice literature, arguing why empowering leadership can decrease employee silence. This study supported the argument that leadership is an important resource for employees to work intrinsically, which supplements the theoretical foundation of the literature on leadership and silence. The results of the mediating effect of intrinsic motivation between empowering leadership and silence not only confirm that motivation is closely associated with employee silence (Morrison, 2011), but also indicate that empowering leadership can build employees’ intrinsic motivation.

Second, this paper directly links the literature on silence and empowering leadership in response to calls to identify additional leadership antecedents to silence/voice beyond transformational leadership (Detert and Burris, 2007; Liu et al., 2010) and ethical leadership (Walumbwa and Schaubroeck, 2009; Walumbwa et al., 2012). Gao et al. (2011) found that empowering leadership can moderate the relationship between trust and voice. Our paper found that empowering leadership has a direct effect on employee silence. The findings suggest that empowering leaders cannot only release employees from being silent because of risk concerns but also intrinsically motivate them to speak up for the benefit of the organization without calculating the cost-benefit equation when they are deciding to make contributions or challenge the status quo.

Third, this paper examined the effects of job design on reducing employee silence. The situational strength argument states that jobs characterized by greater autonomy reduce constraints on employee behavior (Mischel, 1977). Job autonomy determines the extent to which the work setting allows employees to control the process of the job and feel that they own the job, which is necessary for offering autonomous support to work with intrinsic satisfaction. To our knowledge, the literature on employee silence/voice has not been especially integrated with job design. Only one study from Tangirala and Ramanujam (2008b) has reported that a sense of personal control will enhance employees’ expectations that their voice will be effective and thus enhance the frequency of voice. However, this paper draws upon SDT to argue that job design can enhance intrinsic motivation about the job (Hackman and Oldham, 1976), and thus reduce employee silence.

### Practical Implications

The study offers several practical implications. First, when leaders wish to obtain critical information or constructional suggestions, empowering leadership should be functional. Empowering leadership is essential to building employees’ intrinsic motivation, and to accomplish this, leaders must offer autonomy, competence, and relatedness support to employees. Employees’ holding back is not formed in a short time, rather they need to feel greater responsibility for the job to bring about improvements. Several papers suggest that employees who are strong in intrinsic motivation will be more likely to display contextual performance and be committed to their occupation (Yousaf et al., 2015). In daily interaction in the workplace, leaders play an important role in helping employees find intrinsic satisfaction from the job. Thus, from top management to middle managers, empowering leadership should be used to collect sufficient knowledge from employees of all levels.

Second, because the results indicate that the empowering leadership-intrinsic motivation relationship is stronger when job autonomy is high, organizations can design jobs with more autonomy for employees. Jobs designed with more autonomy can create an environment in which every employee feels that he or she owns the job and feels responsible for making every effort to perform by not remaining silent about suggestions that can make improvements. These findings should be welcomed by organizations that engage in fostering a supportive work environment conducive to the accomplishment of organizational goals by creating opportunities for employees to participate in management.

### Limitations and Future Research

We acknowledge several limitations to this study and suggest improvements for future research. First, drawing on SDT, we argue that empowering leadership can fulfill the three psychological needs that are the source of intrinsic motivation, but we did not directly measure those needs. Weinstein and Ryan (2010) measured psychological needs when they argued that these psychological needs mediated the relationship between helping and well-being. Future research could measure the three innate needs to determine how they can enhance intrinsic motivation.

In addition, our research found the positive correlation between empowering leadership and employees’ perceived job autonomy. Although in this paper we treated job autonomy as the boundary condition shaping empowering leadership’s effect on intrinsic motivation, future research can further test the relationship whether empowering leadership can directly influence employees’ feelings of job autonomy. It is possible that employees perceive their job autonomy not only based on the job design from their organizations, but also from the way their supervisors manage them on a daily basis.

Furthermore, although China is an important economic player in the world and being understudied in management literature, we collected data in a single organization in China, which may restrict the generalizability of our research findings. While we compensated for this limitation by using multiple firms in the same firm group, future research could test the proposed relationships using a sample consisting of multiple organization settings in different business contexts.

## Ethics Statement

This study was carried out in accordance with the recommendations of Research Guide from Behavioral Science Research Center at Guanghua School of Management. The protocol was approved by the Behavioral Science Ethics Committee at Guanghua School of Management, Peking University, in that we used data obtained from a consulting project and the data were unidentified when we received them. The participants were told that their participation in the survey signified their consent in our use of their responses, in aggregation, for analyses.

## Author Contributions

DJ and LM: theoretical buiding and data analysis. YZ, LM, and RR: theoretical building and data collection.

## Conflict of Interest Statement

The authors declare that the research was conducted in the absence of any commercial or financial relationships that could be construed as a potential conflict of interest.
